# An adolescent girl with syndrome of inappropriate antidiuretic hormone secretion preceding the diagnosis of olfactory neuroblastoma – a case report

**DOI:** 10.3389/fendo.2024.1447685

**Published:** 2024-10-16

**Authors:** Sabitha Sasidharan Pillai, Jerrold L. Boxerman, Jan C. Groblewski, Bradley D. DeNardo, Mohammed K. Faizan, Lisa Swartz Topor, Renee Robilliard, Monica Serrano-Gonzalez

**Affiliations:** ^1^ Division of Pediatric Endocrinology, Department of Pediatrics, Hasbro Children’s Hospital, Providence, RI, United States; ^2^ The Warren Alpert Medical School of Brown University, Providence, RI, United States; ^3^ Department of Pediatrics, Center for Endocrinology, Diabetes and Metabolism, Children’s Hospital Los Angeles, Department of Pediatrics, Keck School of Medicine, University of Southern California, Los Angeles, CA, United States; ^4^ Department of Diagnostic Imaging, Rhode Island Hospital, Providence, RI, United States; ^5^ Division of Pediatric Otolaryngology, Department of Surgery, Hasbro Children's Hospital, Providence, RI, United States; ^6^ Division of Pediatric Hematology-Oncology, Department of Pediatrics, Hasbro Children’s Hospital, Providence, RI, United States; ^7^ Division of Pediatric Nephrology and Hypertension, Department of Pediatrics, Hasbro Children’s Hospital, Providence, RI, United States

**Keywords:** adolescents, children, olfactory neuroblastoma, esthesioneuroblastoma, SIADH, hyponatremia, antidiuretic hormone, eating disorder

## Abstract

**Objectives:**

We present an adolescent in whom olfactory neuroblastoma (ONB) was detected on follow-up magnetic resonance imaging (MRI) 2.5 years after SIADH diagnosis. Our case contrasts prior pediatric reports in which ONB and SIADH were diagnosed concurrently.

**Case presentation:**

A previously healthy 13-year-old girl was found to have SIADH during evaluation for restrictive eating. Work-up ruled out adrenal, thyroid and paraneoplastic causes, diuretic use, and vasopressin receptor and aquaporin channel mutations. Brain MRI was normal except for paranasal sinus (PNS) inflammatory changes to the left fronto-maxillary sinuses and frontoethmoidal recess. The sodium levels normalized with fluid restriction (800-900 ml/m^2^/day). Multiple repeated attempts to liberalize fluid intake resulted in recurrent hyponatremia. Follow-up brain MRIs 4 and 11 months after the initial presentation showed persistent PNS inflammatory changes. A subsequent brain MRI 31 months after initial presentation demonstrated a lesion in the left frontoethmoidal recess extending into the left nasal cavity and biopsy showed low grade ONB. The patient underwent surgery with normalization of serum sodium on liberalized fluid intake. Seven days after surgery, she had recurrence of SIADH, and brain MRI showed remnant of the ONB at the fovea ethmoidalis. She completed adjuvant radiotherapy though her SIADH persisted.

**Conclusions:**

Our case highlights the importance of considering ONB in the evaluation of children with SIADH. Idiopathic SIADH is rare in children and if no cause is identified, computed tomography of sinuses and nasal endoscopy should be considered earlier in the work-up of these patients, particularly in the absence of sinus symptoms.

## Introduction

Syndrome of inappropriate antidiuretic hormone (ADH) secretion (SIADH) is a condition characterized by unsuppressed secretion of ADH from the pituitary or nonpituitary sources (ectopic ADH secretion) or its continued action due to intrinsic activation of the vasopressin receptors in the kidney (hereditary or “nephrogenic” SIADH). SIADH results in euvolemic hyponatremia due to impaired free water reabsorption with less than maximally diluted urine and natriuresis (1, 2). The most common causes of SIADH in children include respiratory diseases such as pneumonia, bronchiolitis and asthma, meningitis, encephalitis, pituitary tumors, post-transsphenoidal surgery, head trauma, and certain medications (1). Paraneoplastic SIADH is often associated with small cell lung cancer and less commonly with non-small cell lung cancer, extrapulmonary small cell carcinomas, neuroendocrine carcinomas, lymphomas, sarcomas, gastrointestinal, genitourinary and head and neck cancers (3). Olfactory neuroblastoma (ONB), representing 3% of nasal and sinus malignancies, can very rarely cause paraneoplastic SIADH (4).

ONB, also known as esthesioneuroblastoma, is a rare neuroectodermal tumor arising from olfactory epithelium in the superior nasal cavity. It can present with paraneoplastic syndrome mediated ADH secretion in about 2% of cases (5). There have been approximately 20 cases of SIADH due to ONB reported in adults (1). SIADH preceded the diagnosis of ONB by months or years in more than three quarters of the cases of ONB described in adults (4). We performed a literature review and identified 6 cases of SIADH due to ONB in children (**Table 1**). In all pediatric cases, the diagnosis of ONB was made concurrently with SIADH (1, 5–9). We present a previously unreported case of an adolescent in whom ONB was detected on follow-up MRI 2.5 years after SIADH diagnosis.

**Table 1:** Reported cases of SIADH due to ONB in pediatric population.

Patient characteristics	Patient 1 (6)	Patient 2 (5)	Patient 3 (7)	Patient 4 (8)	Patient 5 (9)	Patient 6 (1)	Current patient
Year	1980	2015	2018	2019	2021	2021	2023
Age (years)	17	- (teenage)	17	17	6	11.1	13
Sex	Female	Male	Female	Female	Female	Female	Female
Presenting symptoms	Nasal obstruction and epistaxis	Dizziness, vomiting, unstable gait, headache	Emesis, hallucinations	Lethargy, Nausea, pre syncopal symptoms	Not mentioned	Recurrent epistaxis	None
Laboratory and imaging studies at presentation							
Serum sodium (mEq/L)	110	109	110	111	122	128	124
Serum osmolality (mOsm/kg)	–	–		–	–	258	261
Urine sodium (mEq/L)	73	–	93	–	–	234	689
Urine osmolality (mOsm/kg)	–	–	406	–	–	902	89
Imaging CT/MRI 18F-FDG PET/CT	ONB involving right maxillary, sphenoid, ethmoid sinuses, right nasal cavity with extension to nasopharynx & hard palate	Right maxillary sinus ONB with extension into the infundibulum	Left maxillary sinus ONB No metastases	Right maxillary sinus ONB with no metastases	–	Right maxillary sinus ONB with lung metastasis	ONB involving left frontal, ethmoidal and maxillary sinus with prolapse into the left middle meatus of the nose
Time interval between the diagnosis of SIADH and ONB	Concurrently	Concurrently	Concurrently	Concurrently	Concurrently	Concurrently	2 ½ years
Kadish staging	C	B	B	B	B	D	B
Hyams grading			1	1	–	1	1-11
Management	Radiotherapy and chemotherapy	Surgery and radiotherapy	Surgery and radiotherapy	Surgery and radiotherapy	Surgery and chemoradiation	Surgery and chemotherapy	Surgery and radiotherapy

## Case presentation

A 13-year-old girl with no significant past medical history was found to be hyponatremic during evaluation for mild restrictive eating. The patient had lost about 5 kg over a period of 3 months. She endorsed fatigue and muscle cramps. She reported having her menstrual period within the past 4 weeks, but prior to that she had not had her period for 6-8 months. The patient was hospitalized for evaluation and management. Her weight was 50.2 kg (51^st^ %ile, Z= 0.03), height 152 cm (9^th^ %ile, Z= 1.34) and BMI 21.73 kg/m²(75^th^ %ile, Z= 0.66). Serum sodium was 124 mEq/L, serum osmolality 261 mOsm/kg, urine osmolality 689 mOsm/kg, urine sodium 89 mEq/L and copeptin 24 pmol/L (2-26 pmol/L), suggestive of SIADH. She denied diuretic abuse and was not taking medications. She had stable vital signs and unremarkable systemic examination.

**Figure 1:**
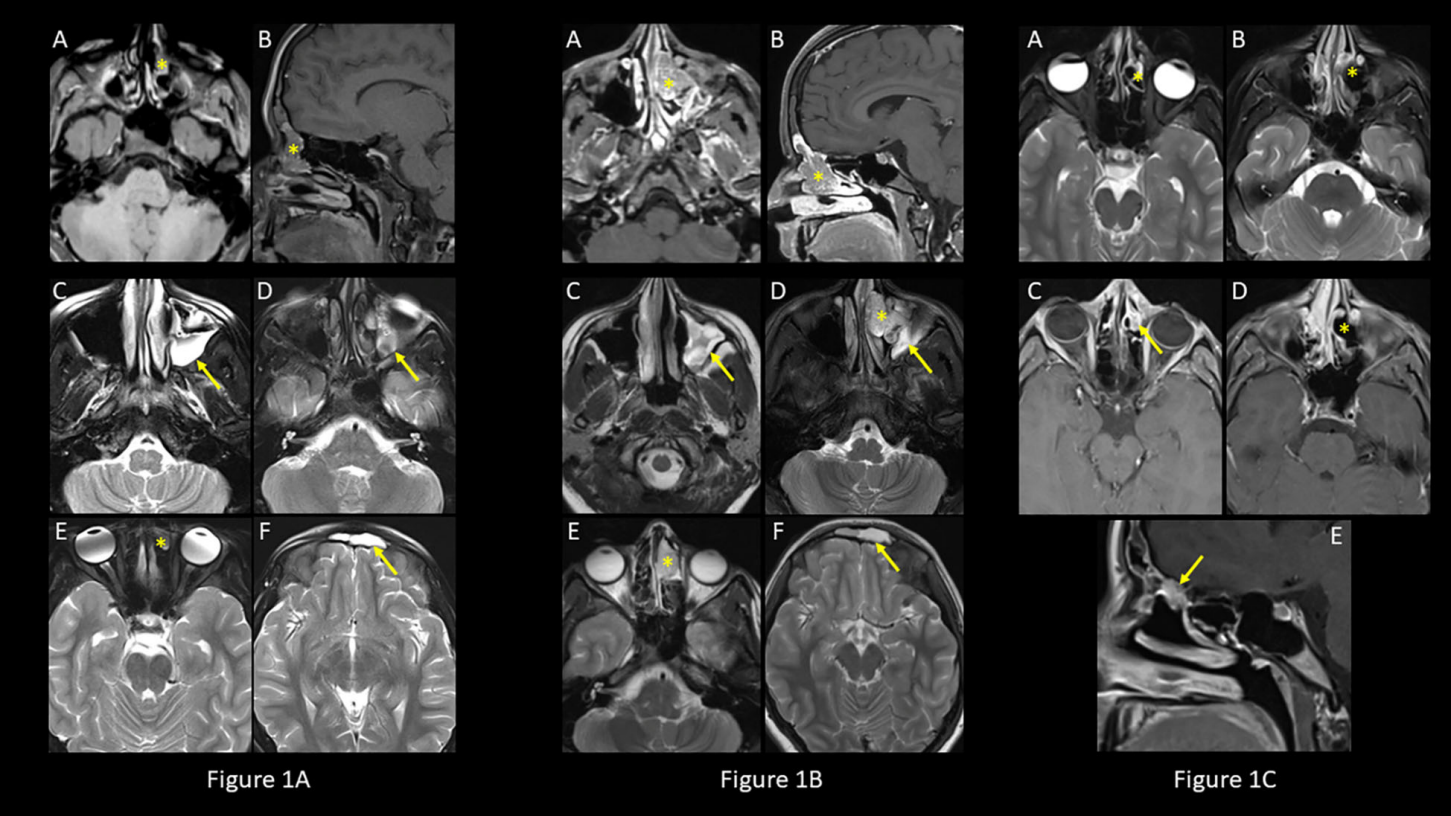
Initial and Follow-up MRI findings. **(A)** Initial MRI exam. Axial **(A)** and sagittal **(B)** post-contrast T1-weighted and axial T2-weighted **(E)** images demonstrate nonspecific enhancing material in left frontoethmoidal sinus (asterisk) with fluid and mucoperiosteal thickening in the maxillary and frontal sinuses on axial T2-weighted images (**C**, **D**, **F**; arrows). **(B)** Follow-up MRI exam, 31 months after the initial MRI exam. Lobulated contrast enhancing mass on axial **(A)** and sagittal **(B)** post-contrast T1-weighted and axial T2-weighted **(D, E)** images (asterisk) with mucoperiosteal thickening and fluid in the maxillary and frontal sinuses on axial T2-weighted images (**C**, **D**, **F**; arrows). **(C)** Post operative MRI exam. Naso-ethmoid resection site (asterisks) demonstrated on axial T2-weighted **(A, B)** and post-contrast T1-weighted **(D)** images, with minimal residual enhancing soft tissue (yellow arrow) in the roof of the nasal fossa at the fovea ethmoidalis on axial **(C)** and sagittal **(E)** post-contrast T1-weighted images (arrow) consistent with persistent tumor remnant in keeping with positive surgical margins.

Further work-up to confirm the diagnosis of SIADH showed a morning cortisol of 15.8 mcg/dL, thyroid stimulating hormone 7.5 uIU/mL (0.35-5.5), free T4 1.0 ng/dL (0.8-1.8), anti-thyroid peroxidase antibody 1282.9 IU/mL (1–60), anti-thyroglobulin antibody 54.3 IU/mL (0-60). She had normal renal parameters (blood urea nitrogen 12 mg/dL and serum creatinine 0.43 mg/dL). Urine screen was negative for toxicology as well as diuretics. She was evaluated for paraneoplastic disease with negative tumor markers including beta-hCG, alpha-fetoprotein, lactate dehydrogenase, urine vanillylmandelic acid and urine homovanillic acid. Imaging included normal abdominal and pelvic ultrasounds and a chest radiograph. Magnetic resonance imaging (MRI) of the brain was normal except for paranasal sinus (PNS) inflammatory changes to the left maxillary sinus, frontal sinuses and left frontoethmoidal recess (**Figure 1A**). Genetic testing for pathogenic variants of the vasopressin receptor and aquaporin channel mutations was negative.

The patient was diagnosed with idiopathic SIADH and started on fluid restriction of 16-20 ounces daily (800-900 mL/m^2^/day) and sodium chloride tablets 1 gram twice daily. Sodium chloride tablets were later discontinued. She was discharged home, remained asymptomatic with normal sodium concentration and did not experience thirst with fluid restriction. Repeated attempts to liberalize fluid intake resulted in recurrent hyponatremia.

Follow-up MRI of the brain at 4 months and 11 months after the initial presentation showed persistent inflammatory changes in the PNS and a pars intermedia cyst. Anterior pituitary screening studies did not reveal any abnormalities. She had another MRI of the brain 20 months later (31 months after the initial presentation) that showed interval enlargement of a lobulated intermediate signal lesion centered at the left frontal ethmoidal recess extending into the left nasal cavity with associated post-obstructive sinus disease (**Figure 1B**). MRI of the face revealed a diffusely enhancing polypoid soft tissue measuring 3.7 cm x 1.4 cm x 1.9 cm, extending from the left inferior frontal sinus through the nasofrontal duct to the anterior ethmoid sinus with prolapse into the superior middle meatus of the nose and left maxillary sinus. Biopsy of the lesion revealed low grade ONB (Hyams grade I-II) (**Table 2**) (10).

**Table 2:** Grading System of olfactory neuroblastoma (10).

Hyams Histopathologic Grading System				
	Grade I	Grade II	Grade III	Grade IV
Lobular architecture preservation	Present	Present	Present/ absent	Present/ absent
Mitotic index	None	Low	Moderate	High
Nuclear polymorphism	None	Moderate	Prominent	Marked
Fibrillary matrix	Prominent	Present	Low	Absent
Rosettes	Homer-Wright rosettes	Homer-Wright rosettes	Homer-Wright rosettes	None
Necrosis	None	None	Rare	Frequent
Kadish Staging System, Modified by Morita				
Stage	Tumor localization			
A	Tumor confined to the nasal cavity			
B	Tumor confined to the nasal cavity and paranasal sinuses			
C	Tumor beyond the nasal cavity and paranasal sinuses, including involvement of the cribriform plate, base of the skull, orbit or intracranial cavity			
D	Tumor with metastasis to cervical lymph nodes or distant metastasis			

Evaluation to stage the tumor including computerized tomography (CT) of the neck/chest and gallium-68 DOTATE positron emission tomography/CT scan revealed no evidence of metastatic disease. She was classified as having Kadish stage B disease (**Table 2**) (10). She did not experience any sinonasal symptoms associated with ONB such as anosmia, epistaxis, or frontal or facial headaches.

The patient underwent endoscopic sinus surgery with endoscopic resection of the neoplasm. Gross resection was achieved but the tumor had to be peeled from the skull base and frontal sinus outflow tract. Pathology revealed microscopic positive margins. Pathology analysis of her resected tumor revealed Hyams grade II ONB with focally positive margins. Immunohistochemical staining for arginine vasopressin could not be done in the resected specimen as it was not clinically available. It would have to be done as a research sample and to be paid for out-of-pocket, which was cost-prohibitive.

The patient had immediate post-operative normalization of serum sodium on liberalized fluid intake, and she regained her thirst sensation, which she had not experienced in a long time. Fifteen days after surgery, she reported recurrent loss of thirst sensation, and biochemical evaluation suggested return of SIADH (serum sodium of 138 mEq/L (135-145 mEq/L), serum osmolality 288 mOsm/L (290-300 mOsm/kg), and urine osmolality 1079 mOsm/L). Post-operative brain MRI (**Figure 1C**) showed minimal residual enhancing tissue in the roof of the nasal fossa at the fovea ethmoidalis, possibly representing persistent remnant of the ONB in keeping with positive microscopic surgical margins and recurrence of her SIADH.

The patient completed adjuvant radiotherapy 3 months post-operatively. Over the 10 months after surgery, the patient reported persistent lack of thirst, with fluid intake between 12-24 ounces per day, and her serum sodium levels ranged between 133 and 138 mEq/L (135-145 mEq/L).

## Discussion

We describe a 13-year-old girl who was diagnosed with ONB on follow-up MRI 2.5 years after the diagnosis of SIADH. She had a detailed work-up including imaging and genetic studies at presentation that failed to detect the cause of her SIADH. Unlike in older adults, idiopathic SIADH is extremely rare in younger patients (11). The patient had serial imaging and frequent follow-up to identify a potential cause for her asymptomatic SIADH, resulting in the detection of ONB at an early stage of the disease on her 4^th^ follow-up MRI of the brain.

ONB can develop at any age, though the incidence usually peaks in the 2^nd^ and 6^th^ decades of life. Patients often present with nonspecific symptoms such as unilateral nasal obstruction, epistaxis, facial pain and headaches, resulting in most patients having an advanced stage of the disease at the time of diagnosis (2). In our patient, the work-up for incidentally detected hyponatremia led to the diagnosis of SIADH, and subsequently to the diagnosis ONB 2.5 years later, prior to the development of sinonasal symptoms associated with ONB.

Kadish-Morita clinical staging and Hyams histopathological grading have been used independently or together to prognosticate and guide treatment decisions for ONB (10). Kadish-Morita staging system has 4 stages A-D; A, ONB confined to the nasal cavity, B, ONB confined to nasal and paranasal sinuses, C, ONB extending beyond the nasal and paranasal sinuses and D, with cervical lymph node or distant metastases (4). Hyams grading system categorizes ONB into 4 grades ranging from grade 1, well differentiated tumor, to grade IV, least differentiated tumor (10). Our patient had Kadish stage B and Hyams grade II disease, indicating low grade, early stage disease despite the prolonged time from identification of SIADH to ONB diagnosis.

Management of ONB includes surgery in conjunction with radiotherapy. Chemotherapy may be indicated, particularly in cases of metastatic disease or unresectable tumor (4). ADH has a short half-life of about 30 minutes in serum and immediate resolution of hyponatremia follows complete resection of the tumor in most cases. Reappearance of hyponatremia soon after resection or in delayed fashion suggests residual tumor or ONB relapse, respectively, as happened in our patient (12). Tumor recurrence can also occur without the development of SIADH. Myers, et al. reported a 79 year old patient with ONB and SIADH treated with palliative radiotherapy who developed brain metastasis 9 months later without SIADH (13). In contrast, Plasencia, et al. reported a patient who developed SIADH with tumor recurrence about 16 years post-excision of initial ONB that had occurred without SIADH (14).

Our literature search identified 6 prior reports of children or adolescents with SIADH due to ONB (**Table 1**). All except one were female with ages ranging from 6 years to 17 years. Symptoms at presentation were available for 5 patients: only 2 had associated sinonasal symptoms, while the remaining 3 had symptoms of hyponatremia. In all prior reported cases, ONB was diagnosed at the time of identification of SIADH, in contrast to our case (1, 5–9). In contrast, SIADH preceded the diagnosis of ONB by months or years in more than three quarters of the cases of ONB described in adults (4). Gabbay, et al. in 2013 observed 25 patients aged ≥18 years from 22 case reports that suggested association between ONB and SIADH: SIADH detected at the time of diagnosis of ONB in 10 (40%), SIADH preceded the diagnosis of ONB by a median interval of 3.5 years in 14 (56%) and SIADH followed the diagnosis of ONB by 2 months in 1(4%) (15). To the best of our knowledge this is the first case report of SIADH preceding the diagnosis of ONB in a pediatric patient.

In the prior reported cases either the work-up for nasal symptoms or the brain imaging done for SIADH led to the diagnosis of ONB. Our patient never had nasal symptoms and her initial MRI findings were negative except for the very mild and non-specific sinus inflammatory changes, even after discussion with radiology. Hence, she was only followed by pediatric endocrinology, and an ENT referral and sinus CT were not performed until the findings on brain MRI were clearer. Even so, ONB was diagnosed early in our patient, as it had a low Kadish score, with no evidence for disease beyond the sinonasal cavity on CT of the sinus/neck/chest and PET/CT.

Our case highlights the importance of considering ONB in the evaluation of children with SIADH. Idiopathic SIADH is rare in younger patients. Hence, if no cause for SIADH is identified, CT nose and sinuses and ENT consultation for endoscopy should be considered earlier in the work-up of these patients, particularly in the absence of the sinus symptoms.

## Data Availability

The original contributions presented in the study are included in the article/supplementary material, further inquiries can be directed to the corresponding author/s.
